# Trust-Enhanced Cloud Service Selection Model Based on QoS Analysis

**DOI:** 10.1371/journal.pone.0143448

**Published:** 2015-11-25

**Authors:** Yuchen Pan, Shuai Ding, Wenjuan Fan, Jing Li, Shanlin Yang

**Affiliations:** 1 School of Management, Hefei University of Technology, Hefei, 230009, Anhui, PR China; 2 Key Laboratory of Process Optimization and Intelligent Decision-Making, Ministry of Education, Hefei, 230009, Anhui, PR China; 3 State Key Laboratory of Computer Architecture, Institute of Computing Technology, Chinese Academy of Sciences, Beijing, 100190, PR China; Beihang University, CHINA

## Abstract

Cloud computing technology plays a very important role in many areas, such as in the construction and development of the smart city. Meanwhile, numerous cloud services appear on the cloud-based platform. Therefore how to how to select trustworthy cloud services remains a significant problem in such platforms, and extensively investigated owing to the ever-growing needs of users. However, trust relationship in social network has not been taken into account in existing methods of cloud service selection and recommendation. In this paper, we propose a cloud service selection model based on the trust-enhanced similarity. Firstly, the direct, indirect, and hybrid trust degrees are measured based on the interaction frequencies among users. Secondly, we estimate the overall similarity by combining the experience usability measured based on Jaccard’s Coefficient and the numerical distance computed by Pearson Correlation Coefficient. Then through using the trust degree to modify the basic similarity, we obtain a trust-enhanced similarity. Finally, we utilize the trust-enhanced similarity to find similar trusted neighbors and predict the missing QoS values as the basis of cloud service selection and recommendation. The experimental results show that our approach is able to obtain optimal results via adjusting parameters and exhibits high effectiveness. The cloud services ranking by our model also have better QoS properties than other methods in the comparison experiments.

## Introduction

With the development of Internet and information technology, cloud computing becomes the core of the new generation computing paradigm. Information services based on cloud computing are increasingly vital and popular in smart city building, operation, and development because of low infrastructure input and high configuration efficiency. Effective selection and recommendation models, which offer optimal cloud services to users, are in high demand for the increasing number of cloud services on the cloud platform.

Traditional recommender systems in e-commerce usually take similarity computation based on user-item matrices to find similar neighbors for the missing values prediction [1, 2]. The values in these matrices are subjective ratings according to users’ preferences for items, which can reflect similarities of users’ subjective preferences. While in the scenario of service recommendation, the matrices usually record objective parameters of invoked services instead of subjective ratings. However, traditional content-based methods [3] can be only used to analyze objective functional parameters of services, and then recommend an optimal cloud service which satisfies the functional demand for users. But even for the same type of services which have equivalent functions, Quality of Service (QoS) might vary largely, depending on their deployed places, required bandwidth, etc [4]. Therefore, it is of great significance to select the optimal and specific cloud services which has the required level of QoS by users from the cloud service candidates recommended by traditional content-based recommender systems [5].

However, the cloud service selection is a complex problem, as it involves many factors, including the reputation (word of mouth) of cloud services providers, QoS, and etc. QoS which describes the non-functional characteristics of cloud services such as response-time and through, is no doubt an important evaluation factor since it objectively reflects the delivery status of the services and the actual perception of the users [6]. However, the existing methods only depend on the QoS ranking to select cloud services and ignore the trust relationships among users. In the social network, users are usually affected by acquaintances in selections of items and services. For instance, in video on demand (VOD) services, users tend to select similar services as their friends do, resulting in a cluster phenomenon inside the friend circles. Hence, modeling and research of social networking have become a hot topic in recent years, and epidemic model [7,8] and game theory [9,10] are also applied to this area. It is necessary to consider users’ trust relationships for finding optimal cloud services in cloud service selection approaches, but it is usually overlooked by many researchers.

In this paper, we devise a new cloud service selection model, which combines the QoS of cloud service and the trust relationships among the users to predict the missing QoS values and rank the cloud services. Firstly, based on interaction frequency between two users, we propose a trust modeling method to evaluate their mutual trust degree (here we assume that the trust degree between two users are equivalent). Secondly, numerical distance and experience usability are employed to estimate the basic similarities which are only based on QoS values. Thirdly, through utilizing trust degrees to enhance the basic similarities, we succeed in predicting the missing QoS values based on the trust-enhanced similarities. Finally, we rank the cloud services for selection. The experimental results show that our approach is successful in ranking and selecting effective cloud services among massive choices by our model offer better QoS compared to others. We further explore several critical parameters within our model, and prove these parameters have the optimal values.

The remainder of this paper is structured as follows. In the next Section, we review current researches on cloud service selection, QoS, similarities estimation, and trust measurement within social networks, respectively. Then, the trust modeling method is proposed for the measurement of trust degrees. Followed by the trust modeling, we utilize trust degrees to enhance the similarities and predict the missing QoS values for ranking cloud services. Finally, we describe our experimental results and get the conclusions.

## Background

Cloud services have gained rising popularity in recent years by providing various applications which are complex and large scale in the cloud environment [11, 12]. QoS is an important research topic in Cloud Computing and Service Computing [13–16]. In 1999, Xiao et al. gave the definition of the emerging Internet QoS [15], and then Daniel discussed that QoS is a combination of availability, security, response time, and throughput properties [14]. Except for functional QoS requirements, freshness and accuracy of QoS are also extensively studied in recent years [13].

The exponential growth of cloud services make it hard for cloud users to select the most suitable one A number of studies have emerged which focus on cloud service selection issue based on QoS [17]. Chen et al. proposed to combine conflict detection and explanation to solve complex enterprise user requirements of cloud service selection [18]. As for cloud service selection infrastructures, Miranda et al. [19] presented to automate the selection by investigating decision support techniques. Their infrastructure is based on the condition that users should consider complex dependencies and heterogeneous sets of services criteria. Qian et al. [20] proposed a cloud service selection system, which not only considers the deployment cost, but also the location of cloud infrastructures. Considering that cloud service selection is a complex task, Smith et al. [21] designed a novel brokerage-based architecture. Machine Learning [22] is also widely employed in service selection. But to our best knowledge, little work has taken trust relations into account.

Despite all this, personalized recommendation methods are main approaches to solve the information explosion problem and are more efficient in service type recommendation. Collaborative filtering method [2] is widely used in the recommender systems and the commercial field (such as Amazon and ebay). Similar estimation in recommendation process is also studied extensively. Zheng et al. used Pearson Correlation Coefficient to calculate the similarity between two service users [23]. Ding et al. employed Jaccard’s Coefficient to measure the experience usability which is an expression of similarity [6]. Cosine similarity is also used in service similarity computation by Zhang et al. [24]. Nevertheless, these works ignore the trust relationships and have low accuracy for specific services recommendation.

In the social network, trust is an old concept in sociology [25]. Jisang has made tremendous contributions in studying trust transfer [26]. His research of improving trust can be applied to e-commerce, mobile commerce and social networks. Zheng et al. proposed a hybrid trust degree model to describe the trust between two users [27], which ont only not only considers the direct trust and indirect trust between users, but also takes the group trust into account. It also considers the influence of trust attenuated with time.

However, no previous study considers the trust relationship in the social network in selecting cloud services. Thus, we propose a QoS-based cloud service selection model which considers both the trust relationships and the similarities among users.

## Methods and Materials

### Trust Modeling of Cloud Service Selection

In recent years, a growing number of enterprises offer a large number of different kinds of cloud services depending on the open cloud computing environment. For example, Tencent applications platform is a big cloud computing platform on which exist more than one hundred thousand apps up to April, 2015. The cloud services belong to 32 categories, including entertainment, music, movie, news, health, shopping, etc. There are a large amount of cloud services of in each category. Therefore, it is a challenging task to select the optimal cloud services from a set of cloud service candidates. In e-commerce, trust which is considered as a significant factor that affects the customers’ decision-making [28], however, has been ignored in many existing cloud service selection methods.

Research on the social network finds that people tend to communicate with those who have similar characteristics [29], based on the homophily feature of the social network. The more interactions there are, the higher trust degree there is [30, 31]. According to previous work [32], interaction frequency can reflect the trust degree to some extent on chat platforms such as QQ, MSN. Therefore, we can use interaction frequency to measure trust.

#### Trust Modeling

**Fig 1:**
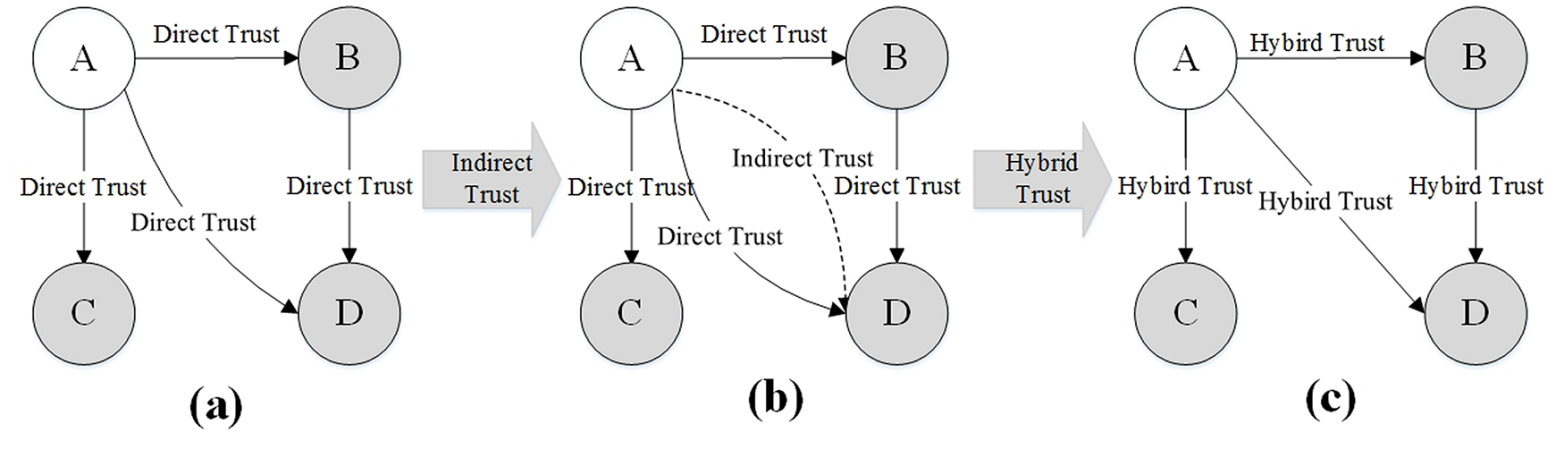
Trust Relationship. (a), (b) and (c) depict direct, indirect, and hybrid trust respectively, where A is the target user. B, C, and D are other users which are trusted by A.

In the social network, users can be represented by nodes and the directed edge from one node to another stands for trust. Since trust relationship is transitive, there is another significant type: indirect trust. Here, we classify the trust into three categories: direct, indirect, and hybrid trust. In Fig 1a, target user A has direct trust with B, C, and D. In Fig 1b, if target user A acquires the trust of D through user B, we define it indirect trust. However, in a real social network, the interactions are much more complex. As presented in Fig 1b and 1c, target user A has both direct trust and indirect trust (through B) to D. Considering all trust paths between two users, it is defined as the hybrid trust.

#### Direct Trust Degree

**Fig 2:**
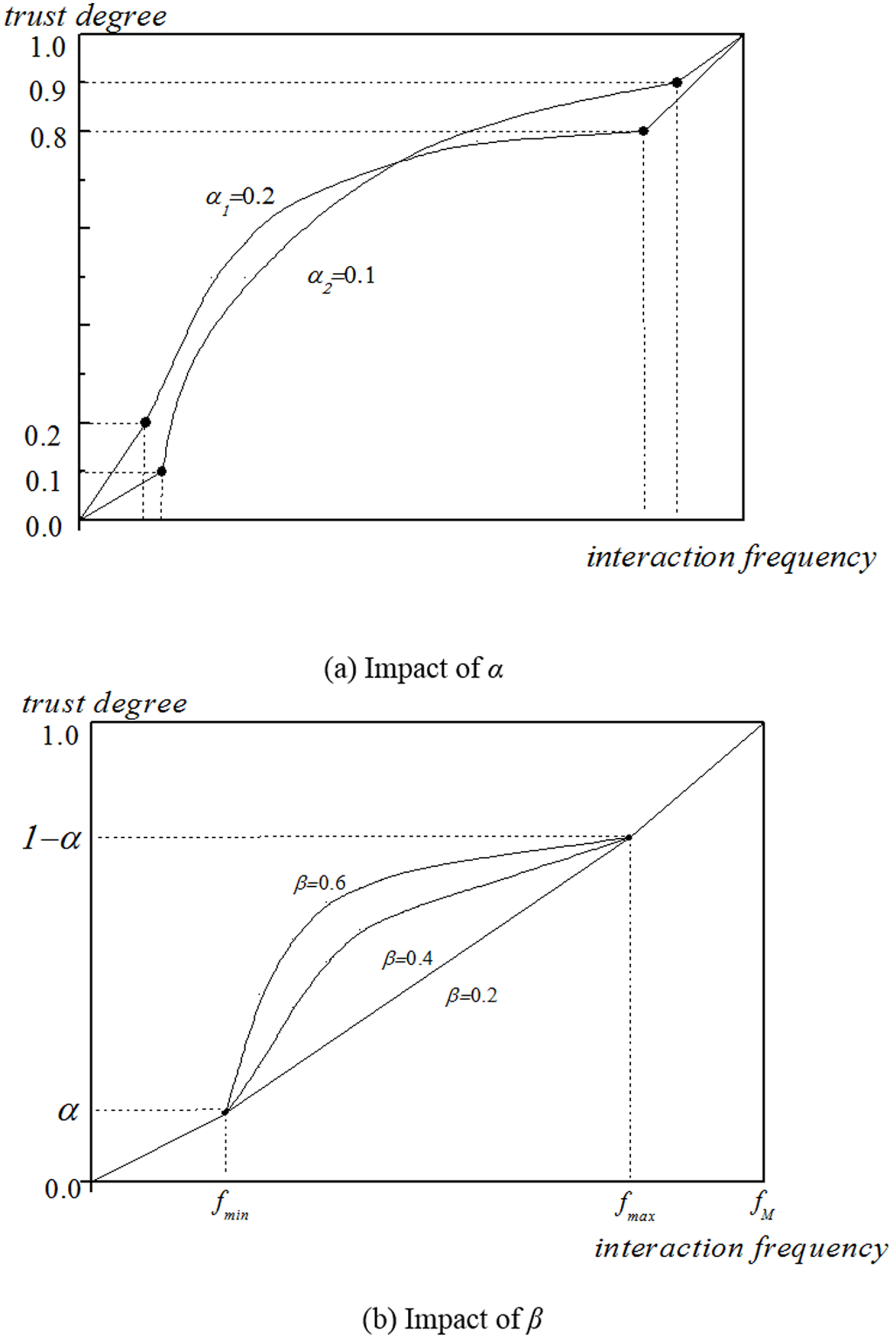
Trust utility function. (a) and (b) depict the trust utility function impacted by parameters *α* and *β*. Suppose the minimum and the maximum trust degrees are 0 and 1 respectively, and by adjusting *α* and *β*, we could change the function form.

Direct trust means that there exists direct interaction between users. It is worth noting that in social psychology, it is not a simple linear functional relationship between the interaction frequency and trust degree. In this paper, we propose a segment trust utility function to explain this relationship. As shown in Fig 2a, trust degree is in the interval of [0, 1] where value 0 and 1 represent the minimum and maximum interaction frequency, respectively. In the segmented function, we set that when the interaction frequency is less than threshold *f*_*min*_ or greater than threshold *f*_*max*_, the trust utility and interaction frequency have a liner function relationship with two different slopes, and while the interaction frequency falls in the interval of [*f*_*min*_, *f*_*max*_], the relationship is represented by a more complex convex function.

The function we propose complies with the law of diminishing marginal utility between *f*_*min*_ and *f*_*max*_. In the economy area, marginal utility measures the additional satisfaction obtained from consuming one additional unit of a good [33]. Diminishing marginal utility is the principle that as more units of a good are consumed, the consumption of additional amounts will yield smaller additional utility. Under the same rule, when the interaction frequency is between the interval of [*f*_*min*_, *f*_*max*_], the increasing rate of trust degree decreases gradually.

Suppose cloud service users are the nodes in the social network and *U* = {*u*_*1*_,…, *u*_*m*_} is the nodes set, with *m* being the total number of the nodes. If two users *u*_*p*_ and *u*_*q*_ interact with each other, there will be an edge connecting them and the length of the edge represents the interaction frequency. The longer an edge between two nodes is, the higher interaction frequency there is. We suppose *f* = {*f*_*1*_,…, *f*_*M*_} is the edge set of interaction frequency in the social network, where *M* is the edge index The elements in *f* are sorted from small to large, with *f*_*1*_ and *f*_*M*_ being the minimum and maximum interaction frequency in *f*. It is clearly that the maximum number of edges is $\frac{m*(m-1)}{2}$, i.e., when every two users have an interaction.

**Definition 1** *α* is the segment-point parameter to adjust piecewise nodes of the trust utility function.

**Definition 2** *β* is the curvature parameter that adjusts the curve curvature of trust utility function.

**Definition 3** *f*_*min*_ and *f*_*max*_ are segment points based on *α*, where *min* = ⎣*M* * *α*⎦ and *mix* = ⎣*M* *(1-*α*)⎦.

As in Fig 2b, when *β* = 0.2 and the interaction frequency falls into [*f*_*min*_, *f*_*max*_], the function is linear. With the increasing value of *β*, the function will curve larger and larger. On the contrary, while adjusting *α*, the function curvature stays the same, but the function segment points move in Fig 2a.

**Definition 4** Trust utility function *t*_*p*,*q*_ used to calculate the trust degree between *u*_*p*_ and *u*_*q*_ is defined as follows:

$$t_{p,q}=\phi \left(x\right)=\{\begin{array}{cc} k_{1}x & 0\leq x\leq f_{min}\\ k_{2}{\left(x+\beta \right)}_{}^{k_{3}} & f_{min}\leq x\leq f_{max}\\ k_{4}x+b & f_{max}\leq x\leq f_{M} \end{array}$$ (1)

**Theorem 1** *k*_*1*_, *k*_*2*_, *k*_*3*_, *k*_*4*_ and *b* are expressed as follows:

$$\begin{array}{l} k_{1}=f_{min}\\ k_{2}=\alpha *\;{\left(f_{min}+\beta \right)}^{\frac{ln\alpha -\text{ln}(1-\alpha )}{\text{ln}(\beta +f_{max})-\text{ln}(\beta +f_{min})}}\\ k_{3}=\frac{\text{ln}(1-\alpha )-ln\alpha }{\text{ln}(\beta +f_{max})-\text{ln}(\beta +f_{min})}\\ k_{4}=\frac{\alpha }{f_{M}-f_{max}}\\ b=\frac{\left(1-\alpha \right)*f_{M}-f_{max}}{f_{M}-f_{max}} \end{array}$$ (2)

**Theorem 1 Proof:**

We use four given points (*0*,*0*), (*f*_*min*_, *α*), (*f*_*max*_,1−*α*), and (*f*_*M*_,1) to solve above parameters in three steps.

Step 1: When *f*_*1*_ *≤ x≤ f*_*min*_, we use (*0*,*0*) and (*f*_*min*_, *α*) with the linear function *k*_*1*_ *x* and get *k*_*1*_ = *f*_*min*_.

Step 2: When *f*_*min*_ *≤ x ≤ f*_*max*_, we use two points (*f*_*min*_, *α*) and (*f*_*max*_,1−*α*) with the curvilinear function $k_{2}(x+\beta ){\;}^{k_{3}}$ and obtain $k_{2}=\alpha *{\left(f_{min}+\beta \right)}^{\frac{ln\alpha -\text{ln}(1-\alpha )}{\text{ln}(\beta +f_{max})-\text{ln}(\beta +f_{min})}}$ and $k_{3}=\frac{\text{ln}(1-\alpha )-ln\alpha }{\text{ln}(\beta +f_{max})-\text{ln}(\beta +f_{min})}$.

Step 3: When *f*_*max*_ *≤ x ≤ f*_*M*_, we use (*f*_*max*_,1−*α*) and (*f*_*M*_,1) with the linear function *k*_4_ *x* + *b*_2_ and get $k_{4}=\frac{\alpha }{f_{M}-f_{max}}$ and $b=\frac{\left(1-\alpha \right)*f_{M}-f_{max}}{f_{M}-f_{max}}$.

Note that except for *α* and *β*, other parameters are considered as constants.

#### Hybrid Trust Degree

In the social network, apart from direct trust, existing research interests focus on indirect trust which usually involves more than one indirect trust path between two users. Hybrid trust is composed of the direct and indirect trust paths. As shown in Fig 1a, target user A trusts D in two paths *r*_1_ and *r*_2_, where *r*_1_ and *r*_2_ denote the direct and indirect paths, respectively. We define *L* as the trust transitivity distance. The *L* of *r*_1_ and *r*_2_ are 0 and 1, respectively.

We put the trust paths from *u*_*p*_ to *u*_*q*_ into a set *Path*_*p*,*q*_. The paths in this set are all indirect trust paths between two users, and the trust transitivity distances are no longer than the maximum trust transitivity distance *L*_*max*_ we set. It can be defined as:

$$\begin{array}{cc} Path_{p,q}=\left\{r_{1},\ldots ,r_{N}\right\} & 0<L_{r_{e}}\leq L_{max},e\in \left\{1,..,N\right\} \end{array}$$ (3)

where *r*_*e*_ is an indirect trust path between *u*_*p*_ and *u*_*q*_. $L_{r_{e}}$ is the trust transitivity distance of *r*_*e*_. *N* is the number of indirect trust paths.

According to (3), we suppose there are *M* users between *u*_*p*_ and *u*_q_ in path *r*_*e*_. The indirect trust degree *t*_*ind*_ *(p*,*q*,*r*_*e*_ *)* between *u*_*p*_ and *u*_*q*_ in path *r*_*e*_ can be calculated as:

$$t_{ind}\left(p,q,r_{e}\right)=t_{p,1}*t_{1,2}*\ldots *t_{M-1,M}*t_{M,q}$$ (4)

To estimate the hybrid trust degree, we integrate the direct trust degree with indirect trust degree. We use the parameter *λ* (*λ*∈[0, 1]) as the weight of direct trust. The hybrid trust degree *T*_*p*,*q*_ can be computed as:

$$T_{p,q}=f(t_{p,q},t_{ind})=\{\begin{array}{cc} \sum_{i=1}^{N}\frac{t_{ind}\left(p,q,r_{e}\right)}{N} & ,L\neq 0\\ \lambda *t_{p,q}+(1-\lambda )*\frac{t_{ind}\left(p,q,r_{e}\right)}{N} & ,L=0 \end{array}$$ (5)

Where *L*≠0 means there is no existing direct trust between two users.

### Cloud Service Selection Process

In this section, we firstly combine multi-QoS properties with the trust degree calculated in the last Section. Secondly, we estimate the basic similarities based on numerical distance and experience usability. Thirdly, we utilize the trust degrees to enhance the basic similarities. Finally, according to use the trust-enhanced similarity, we predict the missing QoS values and select the optimal cloud service.

#### Multi-QoS Properties Combination

QoS is widely adopted to describe non-functional characteristics of services [4]. It is an objective description of the service performance, and is also the expression of the service perceived by the users. As various QoS properties have different dimensions and range of values, we normalize their values to the interval [0, 1] and classify them into two categories: “cost” and “benefit” [5]. If one QoS property is a “benefit” attribute, for example throughput, the higher its value, the more probability for users to choose it.

Suppose *CS* = {*cs*_*i*_ *| i*∈{1,*…*,*n*}} is the set of *n* cloud services. *U* = {*u*_*p*_ *| p*∈{1,*…*,*m*}} is the set of *m* cloud service users. *QoS* = {*Q* ^*k*^ *| k*∈{1,*…*,*l*}} is the set of *l* QoS matrices, with different QoS properties. $Q_{p,i}^{k}$ represents the observed QoS value of the cloud service *cs*_*i*_ invoked by *u*_*p*_ in the matrix *Q* ^*k*^, where *i∈{1*,*…*,*n}*, *p∈{1*,*…*,*m}* and *k∈{1*,*…*,*l}*. According to different attributes of QoS (“benefit” or “cost”), when $Q_{p,i}^{k}$≠ ø, The normalized QoS value $Q_{p,i}^{k}$ can be calculated as following:

$$Q_{p,i}^{k}=\{\begin{array}{ll} \frac{Q_{p,i}^{k}-Q_{\text{min}}^{k}}{Q_{\text{max}}^{k}-Q_{\text{min}}^{k}} & \;,Q_{p,i}^{k}\;is\;"benefit"\\ \frac{Q_{\text{max}}^{k}-Q_{p,i}^{k}}{Q_{\text{max}}^{k}-Q_{\text{min}}^{k}} & \;,Q_{p,i}^{k}\;is\;"cost" \end{array}$$ (6)

where $Q_{\text{min}}^{k}$ and $Q_{\text{max}}^{k}$ are the minimum and maximum QoS values in the *Q* ^*k*^ matrix, respectively.

Since QoS has the feature of multi-property, each of which affects the QoS with varying weight. To support user selection of cloud services, we compose multiple QoS properties on the basis of their weights. *Q*_*p*,*i*_ is the composite QoS value of the multiple QoS properties, and it is defined as:

$$Q_{p,i}=\sum_{k=1}^{l}w_{k}\times \;Q_{p,i}^{k}$$ (7)

where *w*_*k*_ $\left(w_{k}\in \left[0,1\right],\;\overset{1}{\underset{k=1}{\sum }}w_{k}=1\right)$ is the weight of the *k*-th QoS property and thus the value of *Q*_*p*,*i*_ falls into the interval of [0,1]. We assume that if one user invokes one cloud service, this cloud service has all QoS properties values in this invoking process.

#### Basic Similarities Estimation

In traditional e-commerce recommendation systems, Pearson Correlation Coefficient (PPC) has been widely applied to estimate the similarities between different users [1, 2]. Considering the fact that PPC only reflects numerical distance, we combine numerical distance with experience usability [6] to estimate the basic similarities between cloud service users.

The experience usability is calculated based on cloud services invocation historical records [34], which is a significant factor [6] of users’ statistical features. For instance, *u*_*p*_ has invoked *cs*_*1*_, *cs*_*2*_, *cs*_*3*_ and *cs*_*4*_, *u*_*q*_ has invoked *cs*_*5*_, *cs*_*6*_ and *cs*_*7*_, and *u*_*r*_ has invoked *cs*_*2*_, *cs*_*3*_ and *cs*_*8*_. Even though *u*_*p*_, *u*_*q*_ and *u*_*r*_ are strangers in the real world, *u*_*p*_ and *u*_*r*_ have a higher similarity than *u*_*p*_ and *u*_*q*_ or *u*_*q*_ and *u*_*r*_, as they have both invoked *cs*_*2*_ and *cs*_*3*_. We employ Jaccard’s Coefficient [6, 35], which is regularly used to calculate the difference of asymmetric information indicated by binary variables, to estimate the experience usability. Thus the experience usability *J*_*p*,*q*_ between *u*_*p*_ and *u*_*q*_ can be expressed as follow:

$$J_{p,q}=\frac{\left|CS_{p,q}\right|}{\left|CS_{p}\right|+\left|CS_{q}\right|-\left|CS_{p,q}\right|}$$ (8)

where *|CS*_*p*_ *|* and *|CS*_*q*_ *|* are the numbers of cloud services which have been invoked by *u*_*p*_ and *u*_*q*,_ respectively. *|CS*_*p*,*q*_ *|* is the number of cloud services which have been co-invoked by *u*_*p*_ and *u*_*q*_. *J*_*p*,*q*_ falls into the interval of [0, 1], and a higher value indicates that two users have a stronger experience usability to each other.

Given the QoS value *Q*_*p*,*i*_, we can obtain the numerical distance between different users by employing PPC [35] based on user-item regularly [1], which is widely employed in recommender systems for similarity calculation [36]. The numerical distance *N*_*p*,*q*_ between *u*_*p*_ and *u*_*q*_ can be calculated as follow:

$$N_{p,q}=\frac{\sum_{cs_{i}\in CS_{p,q}}\left(Q_{p,i}-{\bar{Q}}_{p}\right)\left(Q_{q,i}-{\bar{Q}}_{q}\right)}{\sqrt{\sum_{cs_{i}\in CS_{p,q}}{\left(Q_{p,i}-{\bar{Q}}_{p}\right)}^{2}}\sqrt{\sum_{cs_{i}\in CS_{p,q}}{\left(Q_{q,i}-{\bar{Q}}_{q}\right)}^{2}}}$$ (9)

where ${\bar{Q}}_{p}$ and ${\bar{Q}}_{q}$ are average QoS values of cloud services invoked by *u*_*p*_ and *u*_*q*_ respectively. *CS*_*p*,*q*_ is the set of cloud services which have been co-invoked by *u*_*p*_ and *u*_*q*_. Then, we combine experience usability *J*_*p*,*q*_ and numerical distance *N*_*p*,*q*_ as follow:

$$Sim_{p,q}^{}=\chi *J_{p,q}+\left(1-\chi \right)\times N_{p,q}$$ (10)

where *χ* is the similarity weight factor, which is in the interval [0,1], and *Sim*_*p*,*q*_ is the basic similarity between users *u*_*p*_ and *u*_*q*_.

#### Trust-Enhanced Similarities Estimation Model

In the social network environment, trust between entities has a huge impact on the decision-makings for the users. Especially in cloud service selection, trust degree will greatly improve the reliability of the selection based on similarity. However, in traditional selection methods, scholars only consider the scores in the user-item matrix and find the similarities among users. In the meantime, trust within these users are ignored. In this paper, we use the trust degree to enhance the basic similarity *Sim*_*p*,*q*_ and obtain trust-enhanced similarity $Sim_{p,q}^{T}$ as following:

$$Sim_{p,q}^{T}=\left[\left(\delta -1\right)*T_{p,q}+1\right]*Sim_{p,q}^{}$$ (11)

**Definition 5** *δ* is the maximum trust-enhanced factor we set. It determines the strength that trust degree can enhance the similarities. The value of *δ* is no smaller than 1.

We can change the value of *δ* to adjust the enhancement strength that trust degree has on the similarities. When *δ* = 1, *T*_*p*,*q*_ has no influence on *Sim*_*p*,*q*_, i.e., we do not take trust into account. When *δ* >1, if *T*_*p*,*q*_ = 0, there is no trust between *u*_*p*_ and *u*_*q*_, ${Sim}_{p,q}^{T}=\delta *Sim_{p,q}$. If *T*_*p*,*q*_ = 1, *u*_*p*_ and *u*_*q*_ have the maximum trust degree, $Sim_{p,q}^{T}=\delta *Sim_{p,q}^{}$. We will explain in the experiment that the upper bound of *δ* is not set here since that the effect of trust enhancement on the basic similarity will not increase unlimitedly with the value of *δ* growing.

#### Cloud Service Ranking Based on Predicted QoS Values

After calculating the trust-enhanced similarities among the cloud service users, we can obtain similar neighbors of the target user by ranking the *Sim* ^*T*^ values. It is worth noting that *Sim* ^*T*^ falls into the interval from–*δ* to *δ*. A positive value indicates similarity and a negative one means dissimilarity. In practice, dissimilar users or less similar users may seriously decrease the effectiveness of the final cloud services ranking. So, we only adopt those users with positive *Sim* ^*T*^ values from the neighbor set and employ the *Top-K Sim* ^*T*^ of users for predicting missing QoS values of the target user as follow:

$$\underset{p\in m,i\in n}{Q_{p,i}^{pre}}={\bar{Q}}_{p}+\frac{\sum_{q\in \varpi_{p}}Sim_{p,q}^{T}\times \left(Q_{q,i}-{\bar{Q}}_{q}\right)}{\sum_{q\in \varpi_{p}}Sim_{p,q}^{T}}$$ (12)

where $Q_{p,i}^{pre}$ is the predicted QoS value of *u*_*p*_ invoking *cs*_*i*_. *ω*_*p*_ is the set of *Top-K* similar users for the target user *u*_*p*_. ${\bar{Q}}_{q}$ is the average QoS value of the cloud services invoked by *u*_*q*_. Finally, we rank the cloud services for *u*_*p*_ in the order of decreasing predicted QoS values for cloud service selection.

## Results

We compare our method with traditional user-based collaborative filtering (user-based CF) and item-based collaborative filtering (item-based CF), both of which only focus on QoS values. We also conduct several experiments to adjust parameters in order to get the optimal solutions and our method turns to be very effective. By exploring three critical parameters, It has shown that our model is able to produce high quality cloud service ranking results.

### Data Sources and Assessment Criteria

#### QoS dataset

Cloud services share some similarities with web services in service properties, particularly in QoS properties. Owing to the lack of the real QoS dataset of cloud services, we used the QoS of web services instead.

In a real world, it is expensive and impossible to invoke thousands of web services for large-scale experiments. We conducted experiments on web service QoS dataset from WS-DREAM team [4]. This dataset includes response-time and throughput QoS values of 5825 real-world web services from 73 countries. These web services are invoked by 339 distributed user-computers located in more than 30 countries from PlantLab, and it is a distributed testbed which contains hundreds of computers in the world. Each of the 339 user-computers invocates all the 5825 web services by sending request to obtain the information of the interactions. Then, the response-time and throughput of invoked web services can be obtained.

In the QoS dataset, throughput and response-time values are in the range of 0–1000 kbps and 0–20 second, of which the mean values are 47.386 and 0.91 respectively. Most of the throughput and response-time values fall between 5–40 kbps and 0.1–0.8 seconds.

#### Trust Simulation

Since there is no suitable real data supporting our trust-modeling, we simulated a social network environment based on our framework in Trust Modeling of Cloud Service Selection Section. This is also the way taken in other experimental evaluations reported in previous studies [37]. In this paper, we use the interaction times to reflect the interaction frequency. For example, *u*_*p*_ and *u*_*q*_ have sent two messages to each other on QQ by now, then the value of interaction frequency between *u*_*p*_ and *u*_*q*_ is equal to two. If every two users interact with each other, the number of interaction times is 57291* (338+…+1). But in the real world, it is impossible that every two random users have interaction relationships. Therefore, we chose 339 users to form the interactive network with the interaction density of 1%, 2%, 5% and 7%, i.e., the number of interacting times are 573 (⌊57291*1%⌋), 1145 (⌊57291*2%⌋), 2865 (⌊57291*5%⌋), and 4010 (⌊57291*7%⌋) respectively. Given the number of interaction times, we simulate and form the trust-network.

**Fig 3:**
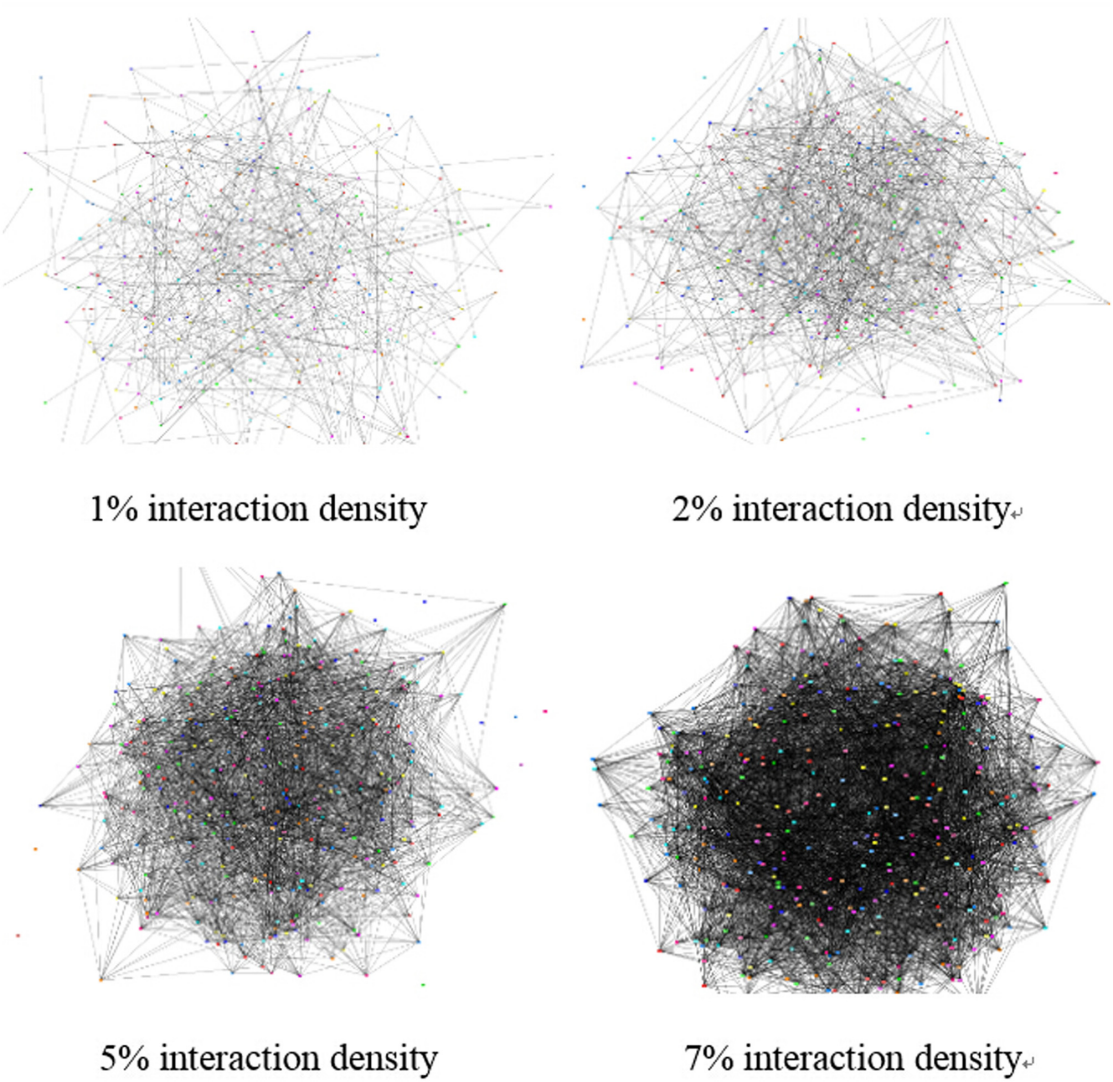
Interaction density. (a), (b), (c) and (d) depict the results of the simulation in 1%, 2%, 5% and 7% interaction density, respectively. Two connected users have an interacting relationship, and the length of the edge indicates their interaction degree.

We generated the trust network in three steps. Take the interaction density of 1% for example. (1) We repeated 573 iterations for generating the value of interaction times, and the iteration times in each iteration falls into the interval [1, 100], and they follow the Gaussian distribution. (2) Our simulation system randomly draws two users from the 339 users and assigns one of the interacting times obtained in (1) as the real interacting situation between them. (3) Repeat (2) 573 times and get the simulated social network. The social network we get through above steps as shown in Fig 3.

#### Assessment Criteria

We assess the effectiveness of our model with Mean Absolute Error (MAE) and Root Mean Squared Error (RMSE), which are defined as:

$$\left\{ \begin{array}{l} MAE=\frac{\sum_{}^{}{}_{p,i}\left|Q_{p,i}-Q_{p,i}^{pre}\right|}{N}\\ RMSE=\sqrt{\frac{\sum_{}^{}{}_{p,i}{\left(Q_{p,i}-Q_{p,i}^{pre}\right)}^{2}}{N}} \end{array}\right.$$ (13)

where *Q*_*p*,*i*_ is the actual QoS value of the cloud service *cs*_*i*_ observed by user *u*_*p*_. $Q_{p,i}^{pre}$ is the predicted QoS value by our model, and *N* is the number of predicted QoS values. Smaller values of MAE and RMSE indicate better results.

We utilize Kendall Rank Correlation Coefficient (KRCC) to calculate ranking similarities between methods with the original ranking (the original ranking is computed based on web service QoS dataset). KRCC has been introduced in many ranking-oriented researches, since it can be easily implemented and has high accuracy. KRCC can be calculated as following:

$$\begin{array}{l} K(u_{p},u_{q})=1-\frac{4\times \sum_{cs\in CS_{p,q}}\tilde{I}\left[\left(Q_{p,i}-Q_{q,i}\right)\left(Q_{p,j}-Q_{q,j}\right)\right]}{\left|CS_{p,q}\right|\times \left(\left|CS_{p,q}\right|-1\right)}\\ \; \end{array}$$ (14)

where *$\tilde{I}(x)=\{\begin{array}{ll} 1, & if\begin{array}{cc} x<0, & \end{array}\\ 0, & \text{otherwise}. \end{array}$* denotes a discordant pair indication function. We employ KRCC to calculate Average Ranking Similarity (ARS) between every two cloud service users as follow:

$$ARS=\frac{\sum_{p=1}^{m}K(u_{p}^{predict},u_{p}^{origin})}{m}$$ (15)

where $K(u_{p}^{predict},u_{p}^{origin})$ is the ranking similarity of cloud service ranking for *u*_*p*_. $u_{p}^{predict}$ and $u_{p}^{origin}$ are based on the selection method and the origin ranking respectively. *m* is the users number. Note that Average Ranking Similarity falls the interval of [–1, 1]. A larger Average Ranking Similarity indicates the service ranking method with better QoS properties.

### Performance Comparison

In this section, we compare the *ARS* of our proposed method with some state-of-the-art approaches.

User-based collaborative filtering: this method adopts PPC to calculate similarities between users and predicted QoS values based on neighbors.

Item-based collaborative filtering: this method adopts PPC to calculate similarities between cloud services and predicted QoS values based on similar services.

We randomly selected and removed 20% data from the web service QoS dataset three times, then, used the remaining 80% data to rank the cloud services and got the following results.

In Table 1, we compare our method with the traditional user-based and item-based methods. Our method is more stable than the others, as the ARS of our method fluctuates the most slightly in these three methods. In addition, all of the ARS values of our method are higher than user-based and item-based methods, which indicates that our cloud services ranking model has better QoS properties.

**Table 1:** Rankings of three methods.

	*Our method*	*User-based*	*Item-based*
First time	0.875	0.742	0.601
Second time	0.901	0.632	0.757
Third time	0.882	0.339	0.498

## Discussions

In order to assess the effectiveness of the parameters, we experimented on three trust-networks in 7% interaction density with 300, 200 and 100 users, respectively. We randomly removed 20% entries from the web service QoS dataset and use the remaining 80% to predict the removed entries. Then, find the optimal parameters through experiments. In our experiment, we set *α* = 0.2 (the segment-point parameter), *λ* = 0.8 (the trust types weight factor), *w*_*1*_ = *w*_*2*_ = 0.5, where *w*_*1*_ and *w*_*2*_ are weights of response-time and throughput respectively, *χ* = 0.5 (the similarity weight factor). We set our parameter *K* to 30, just as [2] did in their former study. We further explored the parameters *β* (curve curvature of trust utility function), *L*_*max*_ (the maximum trust distance) and *δ* (the maximum trust-enhanced factor) to study their impact on predictions.

### Impact of *β*

As we can recall that we measure trust degree using the trust utility function based on interaction frequency, and when the interaction frequency falls into a specified range, the function is convex. *β* is the parameter that adjusts the curvature of the function. We adjusted the value of *β*, i.e., the curve curvature. With the value of *β* adjusting, the impact extent of additional interaction frequency on the trust degree changes.

**Fig 4:**
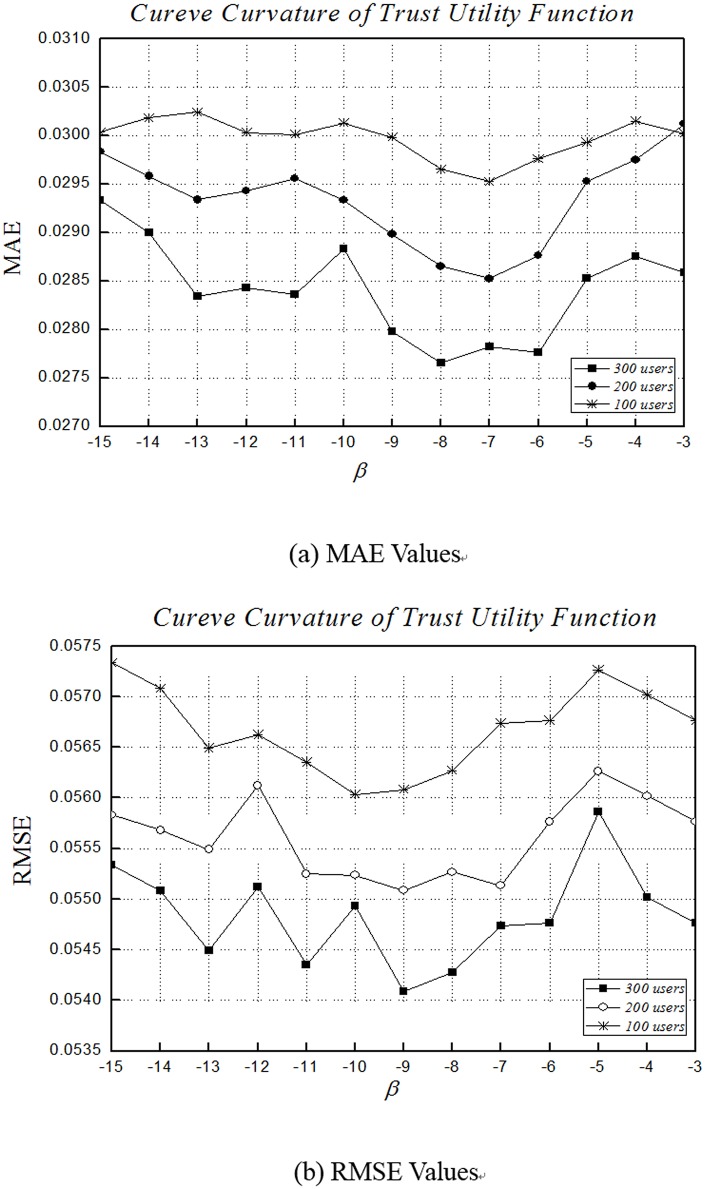
Impact of β of accuracy. (a) and (b) depict the MAE and RMSE fractions of 100 users, 200 users and 300 users for QoS values at the fixed expectation *L*_*max*_ = 2 and *δ* = 2, where the parameter *β* is varied from -15 to -3 in increment of 1.

In Fig 4a, while *β* increases, the curve curvature decreases and the function becomes liner when *β* = -3. As shown in Fig 4a, for the same value of *β*, MAE decreases progressively with the number of users increasing. For the trust network of 300 users, MAE decreases gradually when *β* falls between -15 and -9. When *β* falls between -9 and -6, MAE fluctuates slightly, and reaches an optimum at *β* = -8. When *β* is larger than -6, MAE increases sharply. As described in Fig 4b, RMSE follows the similar trend as MAE, but with larger fluctuations.

Obviously, while the three curves in Fig 4 present similar trends, the results are better with larger user numbers. We show that our trust utility function is better than the simple liner function, and confirm that interaction frequency and trust degree are usually not liner relationship when the interaction frequency is in the interval of specific values. As the curve curvature increases, the results are better and reach the optimum value. The function curve is smoother near the optimal result. After that, the experimental results deteriorate rapidly. It shows that the curve curvature cannot be increased without limit.

### Impact of *L*_*max*_

*L*_*max*_ is the maximum trust distance, which decides the trust network building. It determines how many intermediate users are allowed to propagate trust relationship between two strangers in the network. According to the six degrees of separation, every two strangers in the world can be connected with in less than six persons [38]. Therefore, we set *L*_*max*_ to 0, 1, 2, 3, 4 and 5. Specifically, *L*_*max*_ = 0 means this experiment does not considers indirect trust.

**Fig 5:**
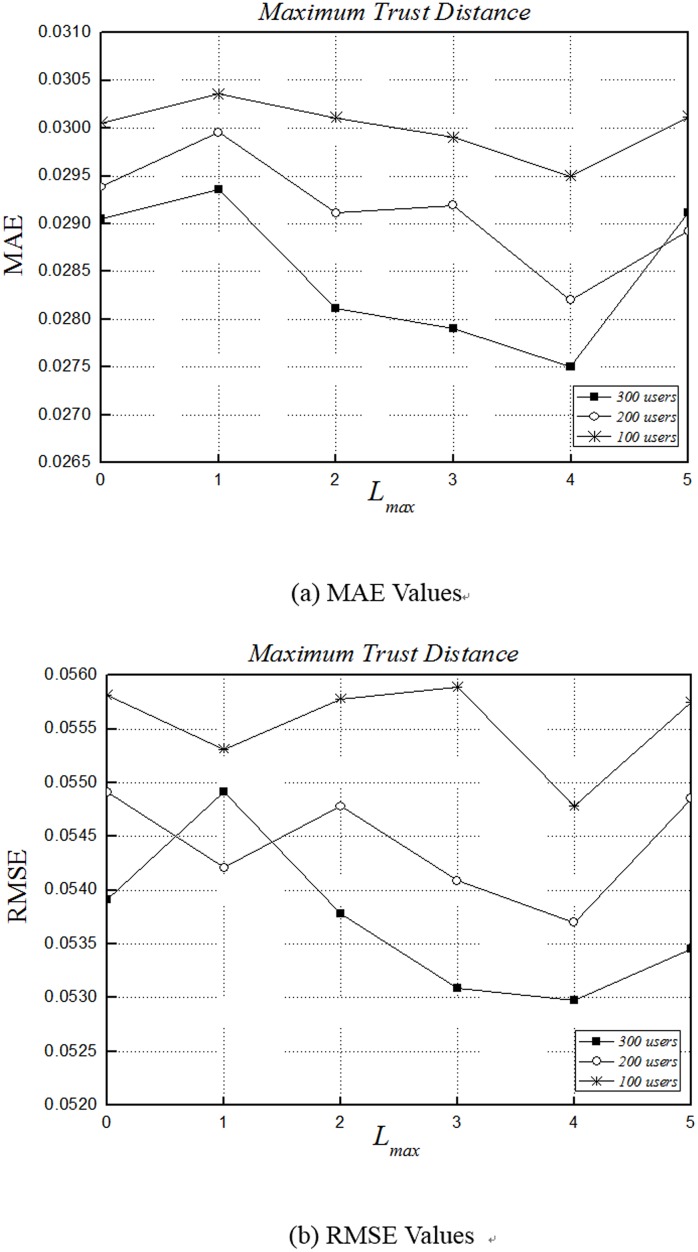
Impact Lmax of accuracy. (a) and (b) depict the MAE and RMSE fractions of 100 users, 200 users and 300 users for QoS values at the fixed expectation *β* = -8, which is the optimum value obtained in the previous experiments, and *δ* = 2. The parameter *L*_*max*_ is varied from 0 to 5 in increment of 1.

Fig 5 shows the experimental results of varying parameter *L*_*max*_. As the number of users increases, MAE shows a decreasing trend. With 300 users, MAE decreases continually while *L*_*max*_ is smaller than 4. When *L*_*max*_ = 4, our results have the best value. Fig 5b shows that RMSE curvilinear trend are similar to MAE, and it also reaches an optimal value at *L*_*max*_ = 4.

These observations indicate the results are better with the number of users increasing, and using indirect trust relationships has better results than only using direct trust. Although the predicted results are better as the maximum trust distance increases at first, but an oversized trust distance harms our prediction accuracy instead. As Fig 5 shows, when the maximum trust distance is equal to 5, the accuracy drops significantly. Therefore, while modeling trust relationships within a network, the maximum trust distance should be set with care in order to obtain the highest accuracy.

### Impact of δ

*δ* is the maximum trust-enhanced factor which determines the strength that trust degree has on the similarities. It is the most important parameter in our method, which controls the influence of the trust relationships.

**Fig 6:**
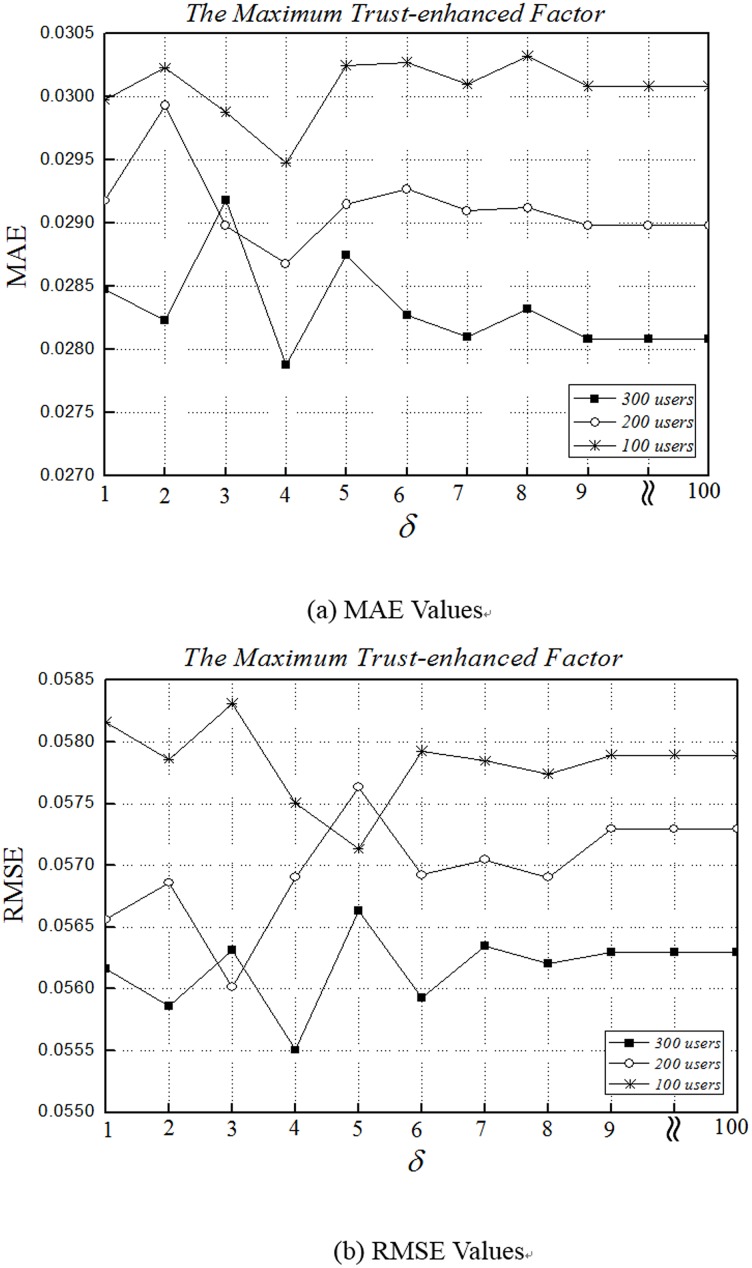
Impact δ of accuracy. (a) and (b) depict the MAE and RMSE fractions of 100 users, 200 users and 300 users for QoS values at the fixed expectation *β* = -8 and *L*_*max*_ = 4, which are the optimum values obtained in the previous experiments. The parameter *δ* is varied from -15 to -3 1 to 100 at a step of 1. When *δ* = 1, we do not consider trust relationships.

As presented in Fig 6, when the number of users increases, both MAE and RMSE decrease drastically. With 300 users, the MAE and RMSE fluctuate sharply between 1 and 8, reaching an optimum value at *δ* = 4. When *δ* is larger than 9, the MAE and RMSE become steady. But all results are worse than *δ* = 4.

Fig 6 proves the effectiveness of utilizing a trust-enhanced similarity, as both MAE and RMSE have a better value at *δ* = 4 than that when *δ* = 1. Nevertheless, the benefit of trust relationship is not unlimited, since MAE and RMSE remain steady when *δ* is larger than 9.

## Conclusions

In this paper, we propose a trust-enhanced cloud service selection model which considers trust relationships in the problem of QoS values prediction. We combine various types of QoS properties together and use the trust degree to enhance the basic similarities. The basic similarities integrate the numerical distance and experience usability. Then, we predict the missing QoS values based on the trust-enhanced similarity, and by virtue of the predicted QoS values, the cloud services can therefore be ranked. Our cloud service selection model has proved to be effective in obtaining better QoS properties than others in the experiments. Finally, we further explore the space of three critical parameters and find the optimal parameters values for our model.

The model we propose can effectively solve the problem of information explosion in cloud service selection, especially when users are faced with massive similar choices. With the advent of the big data era, traditional recommendation and selection methods are not strong enough for massive data analysis and processing and therefore in future work, we will focus on statistical forecasting methods to predict the missing QoS values in big data environment. Especially, intelligent optimization algorithms can be used in the process of cloud service selection, such as particle swarm optimization [39,40] and shortest paths transportation optimization methods [41].

## Supporting Information

S1 Dataset (ZIP)
